# *Eurotium Cristatum* Fermented Okara as a Potential Food Ingredient to Combat Diabetes

**DOI:** 10.1038/s41598-019-54021-4

**Published:** 2019-11-26

**Authors:** Li Yan Chan, Masaki Takahashi, Pei Jean Lim, Shinya Aoyama, Saneyuki Makino, Ferdinandus Ferdinandus, Shi Ya Clara Ng, Satoshi Arai, Hideaki Fujita, Hong Chang Tan, Shigenobu Shibata, Chi-Lik Ken Lee

**Affiliations:** 1 International Food and Water Research Centre, Waters Pacific Pte Ltd, Singapore, Singapore; 2 Organization for University Research Initiatives, Waseda University, Singapore, Singapore; 3 Food Innovation & Resource Centre, Singapore Polytechnic, Singapore, Singapore; 4 Department of Endocrinology, Singapore General Hospital, Singapore, Singapore; 5 Faculty of Science and Engineering, Waseda University, Tokyo, Japan; 6 Division of Chemistry and Biological Chemistry, School of Physical and Mathematical Sciences, Nanyang Technological University, 21 Nanyang Link, Singapore, 637371 Singapore

**Keywords:** Nutrition, Type 2 diabetes

## Abstract

Type 2 diabetes mellitus (T2DM) is a chronic disease, and dietary modification is a crucial part of disease management. Okara is a sustainable source of fibre-rich food. Most of the valorization research on okara focused more on the physical attributes instead of the possible health attributes. The fermentation of okara using microbes originated from food source, such as tea, sake, sufu and yoghurt, were explored here. The aim of this study is to investigate fermented okara as a functional food ingredient to reduce blood glucose levels. Fermented and non-fermented okara extracts were analyzed using the metabolomic approach with UHPLC-QTof-MS^E^. Statistical analysis demonstrated that the anthraquinones, emodin and physcion, served as potential markers and differentiated *Eurotium cristatum* fermented okara (ECO) over other choices of microbes. The *in-vitro* α-glucosidase activity assays and *in-vivo* mice studies showed that ECO can reduce postprandial blood glucose levels. A 20% ECO loading crispy snack prototype revealed a good nutrition composition and could serve as a fundamental formulation for future antidiabetes recipe development, strengthening the hypothesis that ECO can be used as a novel food ingredient for diabetic management.

## Introduction

Type 2 diabetes mellitus (T2DM) is a chronic disease associated with increased morbidity and mortality. It is affecting mankind globally and is one of the most challenging health problems in the 21^st^ century. Singapore is ranked the second among developed countries with diabetes prevalence for aged 20–79 years old. In a study done by the International Diabetes Federation in 2017, 425 million adults aged 20–79 years old are suffering from diabetes, with 606,000 being Singaporeans^1^. By the year 2045, there may be 629 million diabetic adults worldwide with this rising trend. These alarming figures have made diabetes the most pressing national health issue, especially in Singapore. It implicates not just the personal welfare, but also the global macro-economy^1^. Although drug therapy is needed by most T2DM patients, dietary modification is an important component of effective disease management. In this regard, several studies have demonstrated that increasing the intake of dietary fibre can improve glycemic control^2,3^.

In the recent years, there is a growing interest of using okara to improve the blood glucose levels in T2DM patients^4–6^. Okara is the fibrous residue that remains after soymilk and beancurd production processes. Typically, close to 1.2 tons of wet okara are produced from 1 ton of soybean processed for tofu. This makes okara a cheap source of fibre-rich food. Okara, which is packed with a significant amount of proteins, isoflavones, mineral elements, *etc*., is normally used as animal feeds or disposed as waste due to its high perishability, undesirable flavour and grittiness in texture attributes^7–9^. Valorization can be a highly desirable method to utilize the untapped precious nutrients from okara, and more importantly, it can serve to eliminate the economic and socio-environmental problem caused by this waste disposal. Fermentation is one strategy to improve the flavour and texture of okara for food applications^10^. Moreover, the microbial biotransformation of okara can cause a major reduction in the glucoside, malonylglucoside and acetylglucoside isoflavones along with a significant increase of aglycone isoflavones content. The aglycone isoflavones (daidzein, genistein and glycitein) are absorbed faster and in higher amounts than their glucosides in humans, and hence the increase of bioavailability of these isoflavones will potentially improve the antidiabetic effects^11,12^. Liu *et. al*. used *Yarrowia lipolytica* yeast to ferment okara and the fermented product had a greater amount of umami-tasting substances, a cheese-like odour, improved digestibility and enhanced the antioxidant capacity^13^. Chen *et. al*. found that the metabolomic composition and antioxidant activity were improved after fermenting okara with *Rhizopus oligosporus* and *Lactobacillus plantarum*, with *R. oligosporus* displaying more promising results for future development into a functional animal feed^14^. Nonetheless, there are limited research available to show the efforts in producing fermented okara with the enhanced ability to reduce blood glucose levels.

Fuzhuan brick tea, native to the Hunan province, is a type of dark tea fermented dominantly by *Eurotium cristatum*. It was reported that the consumption of Fuzhuan brick tea led to effective improvement of gut health^15–17^, and hence, we postulated that *E. cristatum* will be a suitable microbial candidate. To the best of our knowledge, no research has been done on the fermentation of okara using *E. cristatum*. In Japan, koji mold (*Aspergillus oryzae*) is widely used in the tradition making of Japanese sake, soy sauce, miso, *etc*., where the antidiabetic effects of *A. oryzae* fermented soybean are well studied^18–20^. The application of okara koji, okara fermented by *A. oryzae*, as a partial flour replacement in the preparation of cookies and cupcakes was established by Matsuo in 1999^21^, but the research done on blood glucose lowering effects of okara koji is limited. *Mucor racemosus Fresenius* is involved in the production of sufu, which is a fermented cheese-like soybean product in China and Vietnam, that existed as a delicacy way back to the Wei Dynasty (220–265 AD)^22^. Though Yu *et. al*. demonstrated the enhancement of okara after fermenting with *M. racemosus*, the results focused more on the physical attributes instead of the possible health benefits^23^. *Lactobacillus delbrueckii* subsp. *bulgaricus* is generally used alongside with *Streptococcus thermophilus* as a yoghurt starter culture, and Tu *et. al*. managed to convert okara’s insoluble dietary fibre to soluble form by using this culture mix. Fermented okara can be used as prebiotics to modulate the growth of beneficial gut microbes that confers health benefits^24^ and the ability of soy-okara yoghurt to improve obesity^25,26^ has been demonstrated.

Food and metabolomics have a close relation and has existed in food science for at least over a decade^27^. Interestingly, while food scientists and bioengineers have intensively carried out research on food composition, quality, safety, preservation, and physical attributes, they have often neglected the human or clinical studies of the food-related molecules they have discovered. Medical doctors and clinical researchers adopt the reverse trend. Foodomics is a new concept to bridge the gaps between this interdisciplinary research by integrating the medical and food sciences innovation. Among the omics system technologies, metabolomics is one of the most prominent platforms of analysis^28^.

Herein, we report four food safe microbial fermentations using tea (*E. cristatum*), sake (*A. oryzae*), sufu (*M. racemosus*), and yoghurt (a mixture of *L. d. bulgaricus* and *S. thermophilus*), and discuss how fermented okara can be used as a functional food ingredient to reduce blood glucose levels. We will demonstrate how non-targeted metabolomics approach can identify the markers of interest and consequently, *in-vitro* α-glucosidase activity studies and *in-vivo* mice models are presented to support the findings of using the fermented okara as a potential functional food ingredient for diabetic management.

## Results

### α-Glucosidase activity of the ethanolic okara extracts

**Figure 1:**
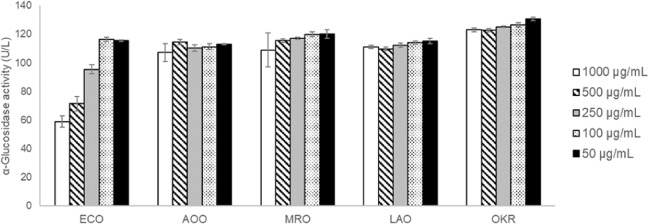
α-Glucosidase activity of fermented okara ECO, AOO, MRO, LAO and non-fermented okara OKR at 50, 100, 250, 500, 1000 µg/mL concentration.

The α-glucosidase enzyme activity was evaluated at various concentration (50, 100, 250, 500, 1000 µg/mL) for the different ethanolic okara extracts. *E. cristatum* fermented okara, *A. oryzae* fermented okara, *M. racemosus* fermented okara or *Lactobacillus delbrueckii* subsp. *bulgaricus* and *Streptococcus thermophilus* fermented okara were labelled as ECO, AOO, MRO and LAO, respectively, whereas non-fermented okara was labelled as OKR. In Fig. 1, the results showed that ECO extracts acted as potential α-glucosidase inhibitor, and displayed the most reduction in the enzymatic activity, as compared to AOO, MRO, LAO and OKR. The efficacy can be observed from 250 µg/mL onwards.

### Metabolomics analyses

**Figure 2:**
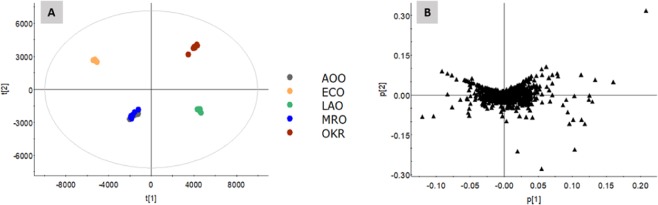
(**A**) PCA score plot of five okara samples in ESI^−^ and; (**B**) Loadings plot in ESI^−^.

Raw data from UHPLC-QTof-MS^E^ were analyzed by Progenesis QI software before exporting into EZInfo software for data analysis. Unsupervised principal component analysis (PCA) was performed to investigate the possible clustering of okara fermented by different microbes for both the negative (ESI^−^) and positive (ESI^+^) ionization modes. In ESI^+^, the discrimination was not obvious (data not shown), hence the subsequent analysis was focused on the data from the ESI^−^ mode. The PCA score plot in Fig. 2, showed separation into distinctive clusters of OKR (control), ECO, AOO, MRO and LAO. The clustering of AOO and MRO, however, were not well-segregated, indicating that the differences between the two fermented okara were not significant.

**Figure 3:**
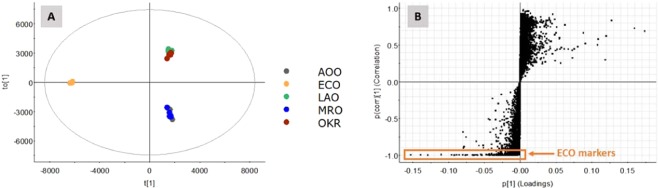
(**A**) OPLS-DA score plot and; (**B**) S-plot of ECO vs the rest.

In order to differentiate ECO from the other types of okara, as well as to identify potential markers that contributed to the reduction of α-glucosidase activity, a supervised multivariate analysis was performed using orthogonal partial least square discriminant analysis (OPLS-DA). OPLS-DA is a supervised technique where the compound ions are classified into the groups using regression and prediction methods. The OPLS-DA scores plot generated from the ESI^−^ ion MS^E^ data for the respective okara samples is displayed in Fig. 3A. A S-plot was created (Fig. 3B), to quickly highlight those characteristic features responsible for the differences found in ECO from the rest. The features with the highest confidence and contribution (highlighted in orange) were selected and imported back into Progenesis QI for verification and further evaluation of the identity. The tentative structural elucidation of some of the compounds that contributed to the discrimination are listed in Table 1.

**Table 1:** Compounds tentatively identified in ECO extract using UHPLC-QTOF-MS^E^.

No	RT (min)	Precursor ion [M–H]^−^	Error (ppm)	Formula	Tentative identification
1	3.04	273.0747	−8.0	C_15_H_14_O_5_	Apiferol
2	3.43	285.0378	−9.4	C_15_H_10_O_6_	Citreorosein
3	3.92	283.0588	−8.4	C_16_H_12_O_5_	Glycitein
4	3.99	271.0588	−8.7	C_15_H_12_O_5_	Rubrofusarin
5	4.91	269.0425	−11.4	C_15_H_10_O_5_	Emodin
6	5.28	285.0372	−11.4	C_15_H_10_O_6_	8-Hydroxygenistein
7	5.59	287.1273	−5.7	C_17_H_20_O_4_	Ethylsuberenol
8	5.95	283.0588	−8.4	C_16_H_12_O_5_	Physcion
9	6.41	221.0796	−10.5	C_12_H_14_O_4_	2,3-Dihydro-3-hydroxy-6-methoxy-2,2-dimethyl-4H-1-benzopyran-4-one
10	6.47	299.0533	−9.4	C_16_H_12_O_6_	Kalafungin
11	7.02	283.1684	−6.8	C_19_H_24_O_2_	Linalyl cinnamate

### Determination of anthraquinones in ECO and ECO_puff

**Figure 4:**
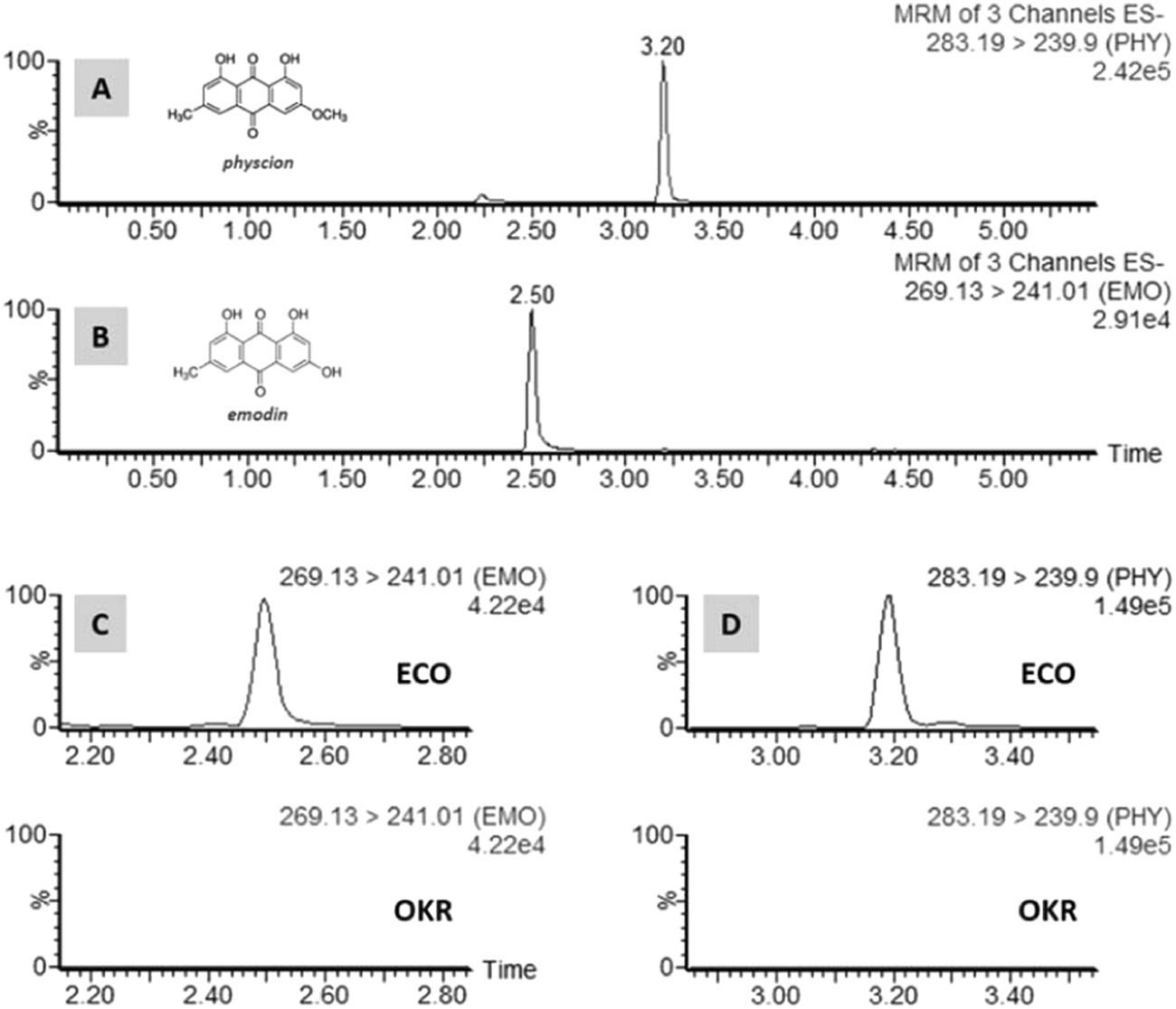
Representative UHPLC-MS/MS chromatograms of pure commercial standards (**A**) physcion and (**B**) emodin. A typical comparison of ECO (top) and OKR (bottom) for (**C**) emodin and (**D**) physcion.

Independent verification of two chosen markers of ECO was done using the UHPLC-MS/MS in MRM mode. These two markers, emodin and physcion, are anthraquinones which have shown potential antidiabetic activity^29,30^. The representative chromatograms of each compounds under the optimized conditions are shown in Fig. 4A,B. Figure 4C,D also clearly illustrated that these two anthraquinones were metabolites by *E. cristatum* fermentation and were absence in OKR. ECO was found to contain 10.20 ppm emodin and 422.42 ppm physcion, respectively. ECO powder was cooked and processed into a crispy snack, named ECO_puff with 20% ECO loading. It was found to contain 1.80 ppm emodin and 50.97 ppm physcion, respectively (Table 2). The OKR extract was spiked with 4 ppm emodin and 400 ppm physcion, which gave 107% and 95% in recovery, respectively.

**Table 2:** The determined concentration of anthraquinones in ECO and ECO_puff.

Compound	Calibration curve	R^2^	ECO (ppm)	ECO_puff (ppm)
Emodin	y = 37.61x −0.13	0.999	10.20 ± 0.13	1.80 ± 0.02
Physcion	y = 6.27e^−4^x^2^ + 1.05x −122.57	0.999	422.42 ± 5.23	50.97 ± 0.30

Data expressed as mean ± SD (*n* = 4). The observed concentrations were based on 50 mg ECO and 250 mg ECO_puff samples, and were expressed as ppm after recalculation.

### The effect of ECO on postprandial blood glucose and blood insulin in mice

**Figure 5:**
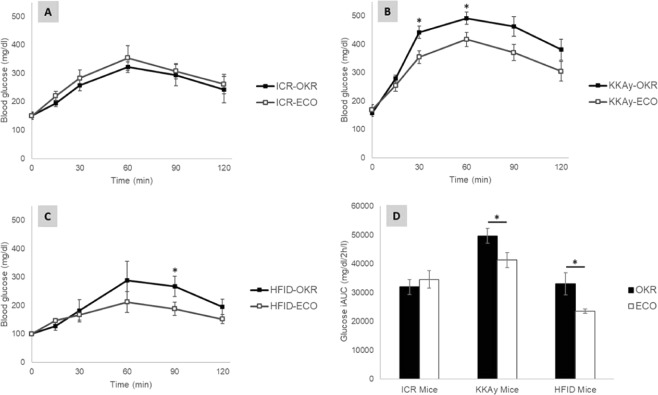
Postprandial blood glucose levels after preload interventions of (**A**) OKR (*n* = 5) or ECO (*n* = 5) in ICR mice model; (**B**) OKR (*n* = 10) or ECO (*n* = 10) in KKAy mice model; and (**C**) OKR (*n* = 10) or ECO (*n* = 10) in HFID mice model. (**D**) iAUC of blood glucose levels for the respective three mice models. Results are expressed as mean ± SE. Asterisks indicate significantly different from the control group (*P < *0.05*).

A preload intervention of okara (OKR or ECO) was conducted 15 min before CS administration. The blood glucose levels were measured at different timepoints over a span of 2 h for three different mice models, (i) the ICR mice model represents healthy/normal individuals; (ii) the KKAy mice model represents T2DM individuals; and (iii) the HFID mice model represents high fat diet induced insulin resistance in ICR mice. The results indicate that ICR mice showed similar blood glucose level patterns after administrating ECO or OKR, and no significant differences were observed (Fig. 5A). For the KKAy and HFID mice models, the blood glucose levels after administrating ECO were significantly lower than OKR treatment. Two-way ANOVA showed that there was a statistically significant effect of time (*P* < *0.01*) and interaction time*okara (*P* < *0.05*) on blood glucose levels in KKAy mice. Post-hoc tests revealed that the blood glucose levels of the KKAy mice after oral administration of cornstarch under the ECO treatment at 30 min (*P* < *0.05*) and 60 min (*P* < *0.05*) were lower than those eating OKR (Fig. 5B). Similarly, two-way ANOVA also showed that there was a statistically significant effect of time (*P* < *0.01*) and interaction time*okara (*P* *=* *0.059*) on blood glucose levels in HFID mice. Post-hoc tests revealed that the blood glucose levels of the HFID mice after oral administration of cornstarch under the ECO treatment at 90 min (*P* < *0.05*) were lower than those eating OKR (Fig. 5C). In addition, the iAUC of blood glucose levels was lower in both the KKAy (*P* < *0.05*) and HFID (*P* < *0.05*) mice after consuming ECO as compared to those consuming OKR (Fig. 5D).

The insulin response was not significantly different between OKR or ECO treatment in both KKAy and HFID mice (Supplementary Fig. 1). Different okara treatments did not change the insulin secretion, while the insulin secretion was generally lower in KKAy mice than in HFID mice.

### Acute oral toxicity of ECO in female rats

All animals in the experimental and control groups showed an increase in the body weight on day-7 and day-14 as compared to day-0 (Supplementary Table 1). However, the increase in body weight was not statistically significant. Overall, oral administration of 2 g/kg bw ECO caused neither abnormalities nor death in any of the rats during the observation period. Consequently, the LD50 value (single dose, oral administration) of ECO is considered to be more than 2 g/kg bw in female rats.

### Proximate composition of OKR, ECO and ECO_puff

The proximate composition of okara before and after fermentation by *E. cristatum* is shown in Table 3. Carbohydrate was consumed during the fermentation, with a significant increase in protein, fat and ash. This information was used to estimate the composition for ECO_puff (Supplementary Fig. 2).

**Table 3:** Proximate composition of okara before and after fermentation by *Eurotium cristatum*.

	per 100 g dry matter		
	OKR	ECO	ECO_puff^a^
Energy	407 kcal	430 kcal	348 kcal
Total carbohydrate	57.2 g	46.3 g	74.6 g
Total dietary fibre	44.4 g	27.6 g	5.5 g
Available carbohydrate	12.8 g	18.7 g	69.1 g
Protein	22.3 g	32.6 g	6.6 g
Fat	9.9 g	12.7 g	2.5 g
Emodin^b^	—	1.02 mg	0.20 mg
Physcion^b^	—	42.24 mg	8.45 mg

^a^Values based on theoretical calculation of 20% ECO loading. ^b^Concentration of emodin and physcion obtained from Table 2.

## Discussion

α-Glucosidase is found in the small intestine and is responsible for the enzymatic hydrolysis of 1,4 – linked polysaccharides, producing glucose as one of the main products. As such, α-glucosidase is a target for the modulation of postprandial hyperglycemia. From our results summarized in Fig. 1, okara fermented by *E. cristatum* produced the largest change in reducing the enzymatic activity of α-glucosidase, and hence may attenuate glucose release after a meal.

Next, we had explored the key marker metabolites pertaining to the fermentation of okara by *E. cristatum* using the metabolomics approach. The PCA plot in Fig. 2A indicated a clear separation of the various okara, except AOO and MRO. Further multivariate statistical analysis by the OPLS-DA and S-plot allowed us to quickly identify the markers in ECO (Fig. 3, Table 1). The anthraquinones (emodin and physcion) were present exclusively in ECO at 10.20 and 422.42 ppm respectively (Table 2). We suggested that the superior performance of ECO might be due to the anthraquinones, which is in accordance to the previous report^29,30^ that demonstrated the antidiabetic potential with potent α-glucosidase and PTP1B inhibitory activity.

In our mice model studies, ECO and OKR (as a control) were explored as the preload intervention to understand the impact on postprandial blood glucose levels after the cornstarch meals in ICR, KKAy and HFID mice (Fig. 5). The postprandial blood glucose levels were significantly lower in the KKAy and HFID mice under the ECO treatment. This demonstrated the importance of fermentation by *E. cristatum* as unfermented okara could not yield the same efficacy. We also found out that the reduction in blood glucose levels were observed without changing insulin levels (Supplementary Fig. 1), and this may have suggested an increase in insulin sensitivity. Postprandial hyperglycemia is a key physiological dysfunction and therapeutic target for T2DM patients. Therefore, this is often recommended as a first line treatment with the greatest opportunity at affordable costs^31^.

The four microbes are originated from traditional fermented food sources. Even though the fermentation of okara using *E. cristatum* is firstly reported here, we believed that the barrier for food safety in this novel ingredient would be low, given that *E. cristatum* has been used in other food matrix. To our delight, ECO showed a good safety profile for consumption according to the acute oral toxicity test carried out in female rats. Nevertheless, continuous efforts will be dedicated to thoroughly investigate the food safety aspects for ECO consumption.

To suit the preload intervention therapy, we suggested a snack food format instead of main meals, such as noodles. A crispy snack (Supplementary Fig. 2) was made using 20% ECO loading which gave a good source of energy (348 kcal), dietary fibre (5.5 g/100 g), protein (6.6 g/100 g), yet low in fat (2.5 g/100 g). It also contained significant amount of emodin (theoretical: 2.04 ppm; actual: 1.80 ppm) and physcion (theoretical: 84.48 ppm; actual: 50.97 ppm) after the cooking process, which was 88.5% and 60.3% of the original ECO powder used (Tables 2 and 3). This shows that ECO is a suitable food ingredient to be used in antidiabetes food recipes. Future works will focus on developing these recipes to balance between the palatability and efficacy.

In conclusion, the current results illustrated a novel food ingredient, ECO, which can be used potentially as a diet intervention for diabetic management. The metabolomics approach has streamlined the whole workflow and facilitated in screening the best performance microbes, and identifying the key functional compounds, such as anthraquinones. Further investigations in healthy and diabetic humans will be reported soon.

## Methods

### Materials and reagents

HPLC grade acetonitrile and methanol was purchased from Fisher Scientific Corporation (Loughborough, UK). All other reagents were of analytical grade. *Aspergillus oryzae* ATCC42149 and *Mucor racemosus Fresenius* ATCC46129 were obtained from the American Type Culture Collection (Manassas, Virginia, US). *Eurotium cristatum* CGMCC3.7934 was obtained from the China General Microbiological Culture Collection Center (Beijing, China). *Lactobacillus delbrueckii* subsp. *bulgaricus* and *Streptococcus thermophilus* fermented okara (LAO) were kindly provided by Prof. Shigenobu Shibata (Waseda University). Emodin and physcion were obtained from Sigma-Aldrich (St. Louis, MO, USA). Fresh okara was a generous gift from a local soymilk stall in Ghim Moh Market and Food Centre (Singapore), and stored at −78 °C before use. *A. oryzae*, *M. racemosus* and *E. cristatum* were maintained and grown on potato dextrose agar (PDA, Sigma-Aldrich) at 28 °C.

### Solid-state fermentation of okara

The precipitates of *A. oryzae*, *M. racemosus* or *E. cristatum*, respectively, were filtered, washed twice, and resuspended into potato dextrose broth to yield about 2 mg/mL culture suspension. Frozen okara was thawed, and autoclaved at 121 °C for 30 min. 300 µL of the culture suspension was added to 20 g of the okara in a petri dish with lid, and mixed evenly with a sterilized spatula. The inoculated okara was incubated at 28 °C for 10 days, and freeze-dried. Non-fermented autoclaved okara (OKR) incubated under the same conditions as the control. *A. oryzae* fermented okara, *M. racemosus* fermented okara or *E. cristatum* fermented okara were labelled as AOO, MRO and ECO, respectively. Methanol (1 mL) was added into 50 mg dried okara sample (ECO, AOO, MRO, LAO or OKR) and the suspension was sonicated at room temperature for 10 min. The mixture was then centrifuged (5000 rpm, 20 °C, 5 min), and 50 uL of the supernatant was diluted with 450 uL methanol. Samples collected were stored at −20 °C before the analyses.

### α-Glucosidase activity

Ethanol (4 mL) was added into the okara sample (ECO, AOO, MRO, LAO or OKR) and the suspension was stirred at 1500 rpm overnight at room temperature. The mixture was then centrifuged (5000 rpm, 20 °C, 5 min) and the supernatant was stored at 4 °C until usage. 75 mM *p*-nitrophenyl α-d-glucopyranoside (100 μL) as the substrate was pre-mixed with the ethanolic okara extract (10 μL) at different concentrations (50, 100, 250, 500, 1000 μg/mL) in a 96-well plate. α-glucosidase (250 U/L, 100 μL) was then added to each well to initiate the enzymatic reaction. The reaction was then allowed to run at 37 °C for 20 min. α-Glucosidase activity was determined by measuring the release of *p*-nitrophenol from the *p*-nitrophenyl α-d-glucopyranoside complex expressed by an increase of absorbance at 405 nm. α-Glucosidase activity of the sample was then calculated relative to the control (no extract) following Eq. 1 below.

1 $${\rm{\alpha }}-{\rm{glucosidase}}\,{\rm{activity}}=\frac{{\rm{Ab}}{s}_{{\rm{20}}}-{\rm{Ab}}{s}_{0}}{{\rm{Ab}}{s}_{{\rm{std}}}-{\rm{Ab}}{s}_{{\rm{H}}2{\rm{o}}}}\times {\rm{125}}({\rm{U}}/{\rm{L}})$$

Abs_20_ and Abs_0_ are the absorbance of the sample at 20 and 0 min, respectively. Abs_std_ and Abs_H2O_ are the absorbance of the control (no extract) and water at 20 min.

### UHPLC-QTof-MS^E^ analyses

UHPLC-QTof-MS^E^ was performed using an ACQUITY I-Class UPLC system connected to a Xevo GS-XS QTof mass spectrometer (Waters Co., Manchester, UK) equipped with an electrospray ion source in the positive and negative ionization mode. The column used was an ACQUITY UPLC BEH C18 column (100 mm × 2.1 mm i.d., 1.7 μm, Waters Co., Milford, USA). Column temperature was maintained at 40 °C for all analyses. The optimal mobile phase consisted of a linear gradient system of (A) 0.1% formic acid in water and (B) 0.1% formic acid in acetonitrile: 0 to 1 min, 90% A; 1 to 8 min, 90 to 10% A; 8 to 8.95 min, 10% A; 8.95 to 9 min, 10 to 90% A; 9 to 10 min, 90% A. The flow rate was set to 0.5 mL/min. Injection volume was 5 μL^32^. All the samples were kept at 10 °C during the analysis. High-accuracy MS data were recorded in both positive and negative ionization modes controlled by MassLynx 4.1 (Waters Co., Manchester, UK) using the following parameters – desolvation temperature, 500 °C; desolvation gas flow, 800 L/h; cone gas flow, 150 L/h; cone voltage, 40 V; source temperature, 150 °C; acquisition range, *m/z* 50–1500; scan times, 0.15 s. The MS^E^ data were acquired in centroid mode using ramp collision energy in two scan functions – low collision energy, 4 V; high collision energy ramp, 20 to 35 V. In the positive ion mode, the capillary voltage was set as 2.0 kV while in the negative mode, the capillary voltage was set as 0.5 kV. All analyses were carried out with an independent reference spray via the LockSpray interference. Leucine enkephalin (Waters Co., Manchester, UK) at a concentration of 0.2 ng/mL was used via a lock spray interface at a flowrate of 100 μLmin^−1^ monitoring for positive ion mode (*m/z* 556.2771) and negative ion mode (*m/z* 554.2615) to ensure accuracy during the MS analysis. Lock spray frequency was set at 20 s. For validation, a pooled sample of all the okara samples was prepared as the quality control (QC) sample.

### Data processing and multivariate data analyses

The MS^E^ data files were uploaded onto Progenesis QI software (Nonlinear Dynamics, version: 2.4)^33^. Chromatographic alignment (with additional manual manipulation), data normalization (with normalize to all compounds) and peak picking (with retention time (RT) and mass to charge ratio (*m/z*) data pairs) were performed by Progenesis QI using the pooled QC sample. A three-dimensional matrix was constructed and then exported into EZinfo 3.0 software for multivariate data analyses. Pareto scaling transformation was applied to the data processing before principal component analysis (PCA) and orthogonal partial least square discriminant analysis (OPLS-DA) were performed. Variables of interest were extracted from S-plot constructed with OPLS-DA, were considered as potential markers. These potential markers ions were transferred into Progenesis QI and subjected to further identification with databases of HMDB, ChemSpider, Metlin and FooDB.

### Determination of anthraquinones using UHPLC-MS/MS

UHPLC-MS/MS was performed using an ACQUITY I-Class UPLC system connected to a Xevo TQ-S micro mass spectrometer (Waters Co., Manchester, UK) equipped with an electrospray ion source in the negative ionization mode. The column used was an ACQUITY UPLC BEH C18 column (50 mm × 2.1 mm i.d., 1.7 μm, Waters Co., Milford, USA). Column temperature was maintained at 40 °C for all analyses. The optimal mobile phase consisted of a linear gradient system of (A) 0.1% formic acid in water and (B) 0.1% formic acid in acetonitrile: 0.0 to 0.5 min, 75% A; 0.5 to 4.0 min, 75 to 10% A; 4.0 to 4.5 min, 10% A; 4.5 to 4.55 min, 10 to 75% A; 4.55 to 5.5 min, 75% A. The flow rate was set to 0.5 mL/min. Injection volume was 1 μL. All the samples were kept at 10 °C during the analysis. The ESI parameters were set as follows: source temperature, 150 °C; desolvation temperature, 500 °C; desolvation gas flow, 800 L/h; cone gas flow, 150 L/h. The multireaction monitoring (MRM) transition parameters (major parent ion >daughter ion), cone voltage, and collision energy were optimized by a flow injection using the IntelliStart program of Acquity UPLC console. This was done with a direct infusion of 200 ppb emodin or physcion at a flow rate of 10 µL/min. The optimized cone voltages were 42 V for emodin, and 40 V for physcion. The molecular ions of emodin and physcion were fragmented at collision energies of 26 and 30 eV using argon as collision gas. Ion detection was performed by monitoring the transitions: *m/z* 269.13 → 241.01 for emodin, and *m/z* 283.19 → 239.90 for physcion. Mixed calibration standards were prepared by mixing and serially diluted with methanol to yield the final concentration of 1, 2, 4, 8, 16, 24, 32 ng/mL for emodin; and 100, 200, 400, 800, 1600, 2400, 3200 ng/mL for physcion. MassLynx 4.1 and TargetLynx data processing software were used for the identification and evaluation of phenolic compounds by comparing retention time and *m/z* transitions of commercial standards using established calibration curves.

### Blood glucose monitoring test

All animal experiments were performed and approved in accordance with the guidelines of the Committee for Animal Experimentation at Waseda University (Permission #2018-A017). Eight weeks old male ICR mice, and male KKAy mice were purchased from Tokyo Laboratory Animals Science (Tokyo, Japan). All mice were housed in an animal room under standard conditions of relative humidity (60 ± 5%), temperature (22 ± 2 °C), and 12 h light-dark cycle (lights-on from 08:00 to 20:00). Zeitgeber time (ZT), ZT 0 and ZT 12 are denote as light-on and light-off time. The light intensity at the surface of the cage was approximately 100 lux. ICR mice were provided with a normal diet (EF; Oriental Yeast Co. Ltd., Japan) and water ad libitum before blood glucose monitoring experiment. KKAy mice were provided with an American Institute of Nutrition (AIN)-93M diet and water ad libitum before blood glucose monitoring experiment. Some ICR mice were fed a high-fat diet consisting of 44.9% fat calories (D12451M; RESERCH DIETS Inc., Tokyo, Japan) and 20% sucrose water ad libitum before blood glucose monitoring experiment. On the day of blood glucose monitoring, each types of mice were randomly divided into two groups of 10 mice each based on body weight and fasting blood glucose levels: OKR group or ECO group. After an overnight fasting, the mice were administrated OKR or ECO (0.5 g/kg bw, i.g.) dissolved in carboxymethyl cellulose (CMC) solution. After 15 min, the mice were given cornstarch (CS: 2 g/kg bw, i.g.) and went through 2 h of blood glucose monitoring.

### Blood glucose and blood insulin measurements

Blood samples collected from the tail vein at time interval of 0, 15, 30, 60, 90, and 120 min after CS administration using Glucose PILOT kit (Aventir Biotech, LLC, Carlsbad, CA, USA). This kit offers a glucose concentration range of 20 to 600 mg/dL. The change of glucose concentration was assessed by the incremental area under curve (iAUC), which was calculated by the trapezoidal rule. Blood insulin concentration was measured at 60 min after CS administration using Ultra Sensitive Mouse Insulin enzyme-linked immunosorbent assay kit (Mercodia AB, Uppsala, Sweden).

### Statistical analyses

Two-way analysis of variance (ANOVA) was used to determine the effects of treatment (OKR or ECO), and the change in postprandial blood glucose levels at each time interval. Statistically significant differences were tested by using Bonferroni post-hoc test, and *P* < *0.05* was considered statistically significant. The iAUC values and blood insulin concentration were assessed by using Student’s t-test, and *P* < *0.05* was considered statistically significant. Data analysis was performed using Predictive Analysis Software, version 23.0 for Windows (SPSS Japan Inc., Tokyo, Japan).

### Acute oral toxicity test

The acute oral toxicity test of ECO was performed by the Japan Food Research Laboratories (JFRL, Tokyo, Japan). ECO was mixed with water for injection and homogenized using a homogenizer (KINEMATICA) to make 100 mg/mL test suspension. Female rats of Wistar/ST strain, at an age of 5 weeks, were purchased from Japan SLC, Inc. Before test, they were acclimated to laboratory conditions for about 1 week to verify that no abnormalities were shown in general conditions. They were housed in plastic cages (five animals per cage) under standard laboratory conditions (temperature: 23 ± 3 °C, light-dark cycle: 12/12 hours). Feed (Labo MR Stock diet, Nosan Corporation) and tap water were provided *ad libitum* throughout the experiment.

Female rats were allocated into experimental and control groups each consisting of five rats. The rats were not fed for about 17 hours before administration. After body weight measurement, the animals in the experimental group were orally administered with the ECO suspension at a single dose of 20 mL/kg bw (at a dosage of 2 g/kg bw ECO) using a stomach tube. The animals in the control group were administered with water for injection, as vehicle control, at the same dose. The clinical observation was carried out frequently on the day of the administration and once a day for the following 13 days. The body weight was measured after 7 and 14 days of the administration. The mean body weight values of the experimental group and the control group were assessed for homogeneity of variance by Levene’s test. Since the Levene’s test was not significant, Student’s t-test was applied for the comparison of two groups (*P* = *0.05*). At the completion of the test, all of the rats were sacrificed for necropsy.

### Proximate composition and recipe of ECO crispy snack (ECO_puff)

The composition of OKR and ECO was performed by the ALS Technichem (S) Pte Ltd (ALS, Singapore) using standard in-house or AOAC methods (Supplementary Table 2). ECO_puff dough was made by blending 17.5 g ECO, 65.1 g tapioca starch, 1.5 g salt and 98.0 g of water with a motor hand-held blender for 30 s. The dough was rolled into an elongated shape of 15–18 cm in length, and diameter of 3 cm, before steaming for 60 min. The dough was then cooled overnight in a 4 °C fridge. The chilled dough was sliced to a thickness of 2–3 mm, and dried using a tray dehydrator at 65 °C for 4 h until the moisture content is about 4%. The sliced dough was then puffed using a microwave at 2000 W high power setting for 15 s to yield the crispy ECO_puff. The proximate composition of ECO_puff was calculated based on the ingredients used.

## Supplementary information

ECO Manuscript Supporting Info r1
