# Supplementary material for: Eurotium Cristatum Fermented Okara as a Potential Food Ingredient to Combat Diabetes
Source: Sci Rep. 2019 Nov 26;9:17536. doi: 10.1038/s41598-019-54021-4 (PMC6879572; doi:10.1038/s41598-019-54021-4)
Supplement: Supplementary file 1 — ECO Manuscript Supporting Info r1 [file 41598_2019_54021_MOESM1_ESM.docx]

***Eurotium Cristatum* Fermented Okara as a Potential Food Ingredient to Combat Diabetes**

*Li Yan Chan, Masaki Takahashi, Pei Jean Lim, Shinya Aoyama, Saneyuki Makino, Ferdinandus Ferdinandus, Shi Ya Clara Ng, Satoshi Arai, Hideaki Fujita, Hong Chang Tan, Shigenobu Shibata, Chi-Lik Ken Lee**

**Supplementary Information**


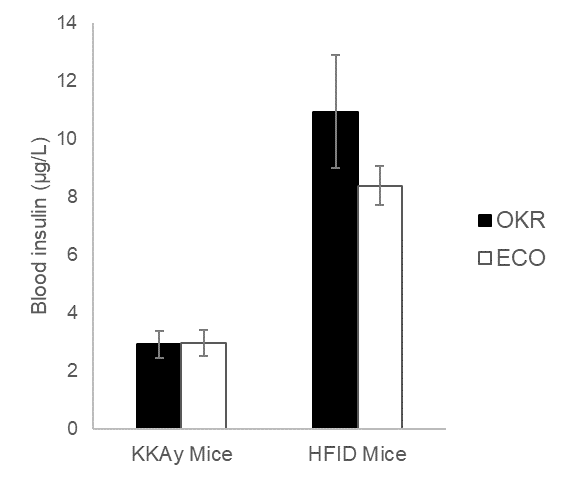


Supplementary Figure 1. Blood insulin concentration measured at 60 min after CS administration. Preload interventions of OKR (n = 6) or ECO (n = 6) were performed in KKAy mice model; and OKR (n = 6) or ECO (n = 5) were performed in HFID mice model.


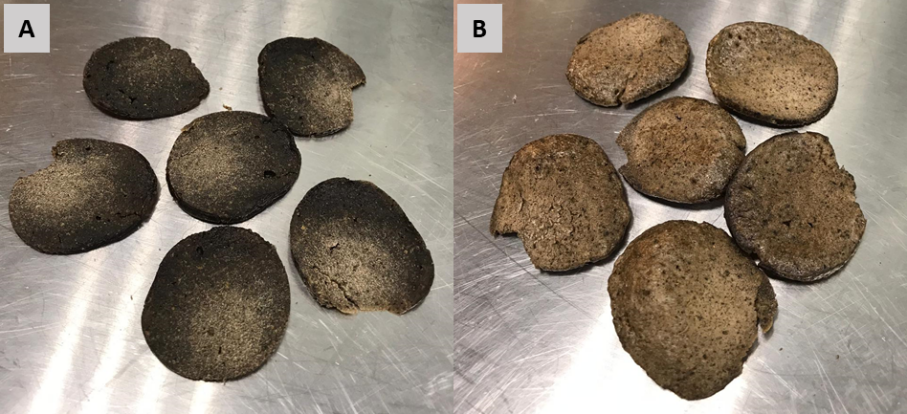


Supplementary Figure 2. Crispy snack made from ECO and tapioca starch; (A) before puffing and (B) after puffing in microwave.

Supplementary Table 1. Body weight of female rats recorded during the acute toxicity study of ECO.

| Group | Body weight (g) | | |
| --- | --- | --- | --- |
|  | Day 0 | Day 7 | Day 14 |
| Experimental | 128.1 ± 6.3 | 162.4 ± 4.6 | 189.3 ± 7.6 |
| Control | 127.9 ± 5.8 | 159.8 ± 8.0 | 181.0 ± 12.0 |

The values are mean ± SD (*n* = 5).

Supplementary Table 2. Testing methods used by ALS Technichem (S) Pte Ltd.

| S/N | Testing Methods |
| --- | --- |
| 1 | Energy Value, Kcal (QWI FD/FC23/By Calculation including Dietary Fibre) |
| 2 | Protein Content, gm (Nx6.25) (Detection Limit: 0.1g/100g)  (QWI FD/FC21/By Kjeldahl Method) |
| 3 | Fat Content, gm (Detection Limit: 0.05g/100g)  (QWI FD/FC22B & OF25, Ref. AOCS Ce 1h-05 (2009)/ GC) |
| 4 | Saturated Fat Content, gm (Detection Limit: 0.01g/100g)  (QWI FD/FC22B & OF25, Ref. AOCS Ce 1h-05 (2009)/ GC) |
| 5 | Trans Fat Content, gm (Detection Limit: 0.01g/100g)  (QWI FD/FC22B & OF25, Ref. AOCS Ce 1h-05 (2009)/ GC) |
| 6 | Cholesterol, mg (Detection Limit: 0.5mg/100g) (QWI FD/FC28/By HPLC) |
| 7 | Carbohydrate Content, gm (Detection Limit: 0.1g/100g) (QWI FD/FC23/By Difference including Dietary Fibre) |
| 8 | Total Sugars as Invert Sugar Content, gm (Detection Limit: 0.5g/100g) (AOAC 923.09 Sec. 44.1.15 (2010)) |
| 9 | Total Dietary Fibre Content, gm (Detection Limit: 0.1g/100g) (AOAC 985.29 Sec 45.4.07 (2010)) |
| 10 | Sodium as Na, mg (Detection Limit: 0.1mg/100g) (QWI FD/FC25/By Acid Digestion/ICP-OES) |
| 11 | Calcium as Ca, mg (Detection Limit: 0.05mg/100g) (QWI FD/FC25/By Acid Digestion/ICP-OES) |
| 12 | Potassium as K, mg (Detection Limit: 0.05mg/100g) (QWI FD/FC25/By Acid Digestion/ICP-OES) |
| 13 | Iron as Fe, mg (Detection Limit: 0.05mg/100g) (QWI FD/FC25/By Acid Digestion/ICP-OES) |
| 14 | Ash Content, gm (Detection Limit: 0.01g/100g) (QWI FD/FC05/By Ashing @ 550 °C) |
| 15 | Moisture Content, gm (Detection Limit: 0.05g/100g) (QWI FD/FC20/By Oven Drying @ 103 °C) |
| 16 | Vitamin D_3_ as Cholecalciferol, µg (Detection Limit: 0.5 µg/100g) (QWI FD/FC117/By HPLC-DAD) |
